# What Does It Mean to Age in Place as an Older Homeless Woman? Facing an Altered Sense of Place, Belonging, and Identity

**DOI:** 10.1093/geroni/igaa057.171

**Published:** 2020-12-16

**Authors:** Judith Gonyea, Kelly Melekis

**Affiliations:** 1 Boston University, Boston, Massachusetts, United States; 2 Skidmore College, Saratoga Springs, New York, United States

## Abstract

The emergence of “aging in place” as social policy in the U.S and globally reflects a deepening understanding that a home is more than a physical domicile, it also represents a source of personal and social identity and offers one a sense of place and belonging. In this qualitative study we explore the question, What does “aging in place” mean to older homeless women navigating the shelter system and streets? Using a phenomenological approach, we conducted semi-structured interviews with fifteen chronically homeless women in their fifties using the shelter system. Our analysis process was inductive and iterative with the culminating phases being the generation and interpretation of themes. Our analysis revealed the links between place, sense of belonging, and identity. To be displaced from a physical home can present challenges to defining one’s very existence. Specific themes emerging from the women’s narratives included the ways in which shelter and street life impacted their sense of personal control, privacy, security, health, and comfort as well as underscored that shelters are dehumanizing places that further diminish one’s sense of self and self-worth. The interviewed women sought to construct a positive sense of self through speaking about their past, present, and future roles as well as identities gained through social relations and place identity connections. Based on the findings, we suggest strategies by which shelters might better respond to unique needs of older women, including adopting ways that do not further disempower or stigmatize them but rather promote pathways out of homelessness.

